# Hospital and Health News

**Published:** 1924-11

**Authors:** 


### HOSPITAL AND HEALTH NEWS.

### Children's Health Lectures.

A "Children's Health" Series of lectures will be delivered at the Institute of Hygiene, 33-34 Devonshire Street, W.1, on Tuesdays at 3.30 p.m., beginning on October 22. The Secretary will be pleased to send complimentary tickets to nurses and those interested in child welfare.

### Honour for a Welfare Pioneer.

The Deptford Borough Council has decided that the name of Miss Margaret Macmillan be recorded on the honours board of the Council Chamber and that she be asked to sign the Borough Roll of Honour in recognition of her work on behalf of the child life of the nation.

### General Nursing Council for Scotland.

The General Nursing Council for Scotland state that in the case of nurses registered before January 1, 1924, a retention fee of 2s. 6d. fell due on September 30, and nurses sending this should enclose their registration certificates to be endorsed.

### "Wildly Utopian."

The Hospitals Conference at Wellington, New Zealand, has passed a resolution asking the Health Department to consider the feasibility of securing the annual medical examination of every person in the Dominion. Some delegates described the proposal as wildly Utopian, but the majority decided on the urgent need for this reform.

### Handsome Tribute to Dr. Brackenbury.

Dr. H. B. Brackenbury, chairman of the Representative Body of the British Medical Association and chairman of the Insurance Acts Committee, has been presented with a canteen of silver, a gold watch and a cheque for £6,500 contributed by members of the medical profession all over the country.

### Health of the Commonwealth.

A course of lectures on "The Health of the Commonwealth" has been arranged by the Royal Institute of Public Health, and is being delivered on Wednesdays at 4 p.m. from October 15 to December 10 in the Hall of the Institute, 37 Russell Square, W.C. 1. The lecturers will include Sir Robert Jones, Professor Lazarus Barlow and Sir Leonard Rogers.

### Six Lectures for Nurses.

A course of six lectures, especially suited to the nursing profession, will be delivered at 6 Guilford Place, Russell Square, W.C. 1, at 3.0 p.m. in the afternoons of November 22, December 6 and 20, January 10 and 24, and February 7 by Miss Mary Chadwick. They will deal with such subjects as "How Psychology May Help the Nurse," "The Nurse and the Staff," etc. The fee for the course of six lectures is 5s.

### An Ideal Medical Centre.

The Presbyterian Hospital, New York, and the School of Medicine of Columbia University have joined forces to establish on Washington Heights, New York City, an ideal medical centre, which will co-ordinate under one administrative control general and special hospitals, colleges, and research laboratories, covering every phase of medical practice and the science of medicine. The ultimate cost may reach £6,000,000 for building alone.

### Appeal Secretary for St. John's, Lewisham.

The appeal for £12,000 for a nurses' home and new wing at St. John's Hospital, Lewisham, has been entrusted to Mr. H. Sheridan-Bickers, who has had experience of publicity and organisation in Canada and the States, besides some journalistic experience in London. Educated at Cambridge, Mr. Sheridan-Bickers was one of the staff employed on the recent Manchester University Appeal Fund, the success of which has been attributed to the propaganda adopted.

### Sheffield Royal Infirmary.

An interesting ceremony took place recently at the Royal Infirmary, when the Board of Management presented to the retiring chairman, Mr. H. H. Bedford, an excellent painting of himself by Mr. Jagger. Many people accepted the Board's invitation to the ceremony, after which tea was served and the hospital was open to inspection. Many items of interest were on exhibition, such as new and old instruments, modern treatment of fractures and skiagrams, showing the condition of the patient before and after treatment.

### Voluntary Boarders in Mental Hospitals.

The City of London Mental Hospital at Stone is the first public mental hospital to accept as voluntary boarders uncertified patients who enter and leave the hospital at their own free will. This, of course, excepts the Maudsley Hospital, which is in a rather different category.

### Winning Plan for a New Hospital.

The Barton-upon-Irwell Union authorities lately secured a site of some thirty acres for a new hospital at Davyhulme. Conditions for designs of the proposed hospital were drawn up by Mr. W. A. Pite, F.R.I.B.A., and the result of the competition is now known. The winning design, which was one of eleven submitted, was that of Messrs. Elcock and Sutcliffe. It affords accommodation for 308 beds, with provision for an ultimate 500, with all the wards on the southern side of the main corridor, where a maximum amount of sunshine and pleasant outlook can be obtained by the patients. The estimated cost is £155,893.

### Successful Effort for Ancoats Hospital.

The Committee and workers of the Bazaar held last May in aid of Ancoats Hospital, Manchester, have presented to Mr. P. M. Oliver, M.P., the Hospital's Treasurer, a sum of £1,061 9s. 1d. as the result of their efforts towards paying for the cost of the new X-ray Department. The Chairman of the Hospital, Mr. William Armitage, has unveiled a tablet in the X-ray Department, placing on record the grateful thanks of the Committee to all those who in any way helped to raise this generous amount.

### Prevention of Tuberculosis.

The Minister of Health is about to make Regulations for the purpose of preventing infection from the employment in and about farms, dairies, etc., of persons suffering from pulmonary tuberculosis. Copies of the draft Regulations which have been prepared for this purpose can be purchased under the description "Draft dated 17th October, 1924, of the Public Health (Prevention of Tuberculosis) Regulations, 1924," from H.M. Stationery Office, Adastral House, Kingsway, W.C. 2, either directly or through any bookseller (price 1d.). Any representations on the subject should be addressed to the Secretary to the Ministry at an early date.

### New Nurses' Home at the Royal Northern.

Princess Louise, Duchess of Argyll, will open the new Nurses' Home of the Royal Northern Hospital, Holloway, on October 30, at 3.30 p.m. The Home is the first section of a larger building, and provides separate bedrooms for 58 nurses, and administrative offices, sitting-rooms, etc., for the complete building, which will eventually accommodate nearly 120 nurses. The building has been provided from money given for this specific purpose, and has been recognised by King Edward's Hospital Fund for London as the most urgent need of the Royal Northern Hospital. The cost, including site, is £33,000, of which £5,900 still has to be collected.

### Enterprise Rewarded.

By means of a four days' bazaar held recently at Buxton —"Ye Olde English Fayre," when the huge area of the hospital under the Great Dome was transformed into an old Derbyshire village of the Tudor period—the Devonshire Hospital, Buxton, has benefited to the surprisingly large extent of £8,000. This building was built over a hundred years ago for the then Duke of Devonshire and was opened in 1859 as a hospital by the generosity of the sixth Duke. To-day it is a national institution, containing 300 beds, for the relief of poor persons suffering from rheumatism and allied diseases, and treating over 4,000 patients annually.

### Flashing the Virtues of the Toothbrush.

Bustling Bermondsey, if we may so style without offence a supposedly drab borough that possesses, among other things, ideas, a club-like and uncommercial Bookshop, a scheme for the sun-cure, and so on, has another plan, too. Its medical officers are understood to be devising electric signs to flash the virtue of the toothbrush at night before the eyes of the inhabitants. This is an extension of the propaganda by leaflet for which the HOSPITAL AND HEALTH REVIEW three years ago held a successful and amusing competition. The three prize leaflets on "The Care of the Teeth" were published in our issue of November, 1921.

### Monkwearmouth and Southwick Hospital.

An appeal, made attractive by concise wording and pleasant architectural drawings of the proposed elevation, has been issued by the Subscriptions Committee of the Monkwearmouth and Southwick Hospital. The plan is to build a new hospital in Newcastle Road on the Ashville site, because the alternative scheme of amalgamation with the sister hospital has not gone through. The North Side, as it is called, has long outgrown its existing hospital accommodation and has not restricted its services to patients in the immediate neighbourhood. The eventual sale of the present building and a sum of £3,000 in hand are expected to provide the equipment. The governors possess the Ashville site. The cost of the building will be £70,000, which the Subscriptions Committee has now begun to raise, with a good start of about £20,000.

### Where an Accident Ward is Most Needed.

The accident ward with twenty beds that is being constructed at the West London Hospital is expected to be ready by Christmas, or in time for the New Year. Hammersmith Broadway seems designed for the scene of accidents, for two main roads out of London complicate the already congested local traffic of the spot. The motor traffic is held to be chiefly responsible, and it is, presumably, two owners of cars who have respectively given £3,000 toward the equipment, and £5,000 conversion loan towards the endowment, of the new ward. This will allow every accident to be treated on the spot, for the present at any rate, but the larger part of the cost of maintenance and equipment has still to be found—perhaps by a voluntary toll given by drivers through the Broadway. Is there no means of collecting on the spot without making more accidents than the proceeds would provide for ?

